# Itaconic acid-modified layered double hydroxide/gellan gum nanocomposites for Congo red adsorption

**DOI:** 10.1038/s41598-022-08414-7

**Published:** 2022-03-14

**Authors:** Shirin Shabani, Mohammad Dinari

**Affiliations:** 1 Chemistry Group, Pardis College, Isfahan University of Technology, Isfahan, 84156-83111 I. R. of Iran; 2 Department of Chemistry, Isfahan University of Technology, Isfahan, 84156-83111 I. R. of Iran

**Keywords:** Environmental sciences, Chemistry, Materials science, Nanoscience and technology

## Abstract

Polymer matrix nanocomposites with nanosized fillers are becoming an inseparable part of various industries owing to their unique properties. Among the nanosized fillers, layered double hydroxides are a good candidate due to their fantastic properties such as the ability to ion exchange and adsorption capacity. In the present work, a simple and green synthesis procedure was applied for the preparation of novel modified Cu–Ca–Al-based layered double hydroxide/polymer matrix nanocomposites. Notably, the synthesis was performed in a water medium as solvent. The layered double hydroxide was synthesized and itaconic acid was used for the surface functionalization of the prepared material. The modified material was then incorporated into the natural polymer of gellan gum to produce polymer matrix nanocomposites with different filler contents. The prepared materials were characterized using infrared spectroscopy, X-ray diffraction analysis, scanning and transmission electron microscopy, X-ray diffraction analysis, thermogravimetric analysis, and N_2_ adsorption/desorption technique. After characterization, the prepared materials were used for the adsorption of Congo red. After investigation of the important experimental parameters, the isotherm and kinetic studies were also performed. Among the studied kinetic models, the pseudo-second-order model and intra-particle diffusion model were obtained the best in the case of Congo red adsorption. The Freundlich isotherm model showed the best results. Finally, maximum adsorption capacities of 80.9, 90.1, and 99.9 mg g^−1^ were obtained for nanocomposites containing 1%, 3%, and 5 wt% of filler, respectively.

## Introduction

Nowadays, water pollution, as an unpleasant result of the industrialization process is a serious international problem. The disposal of toxic substances into the environmental water sources caused severe concerns about the health of creatures^1–3^. Scientists are working to find a solution to this global problem. Several decontamination strategies have been reported to remove toxic substances from environmental media. They are generally included physical, chemical, and biological treatments^4^. Adsorption, coagulation, oxidation, membrane separation, biological degradation, etc. are important methodologies that have been reported^5,6^. As a conventional strategy, the adsorption technique provided several benefits to be used for water treatment. This method is easy to handle, low-cost, and flexible. No harmful by-products are produced during the removal process^5,7^. In the case of organic dyes, various natural and synthetic adsorbents have been reported to be used as adsorbents^8–10^.

Layered double hydroxides (LDHs) consisted of layers with a positive charge and an interlayer region with the ability of anion exchange^11–13^. The stoichiometry of LDHs is demonstrated $\left[ {{\text{M}}_{{1 - {\text{x}}}}^{2 + } {\text{M}}_{{\text{x}}}^{3 + } \left( {{\text{OH}}} \right)_{2} } \right]^{{{\text{x}} + }} \left( {{\text{A}}^{{{\text{n}} - }} } \right)_{{{\text{x}}/{\text{n}}}} \cdot {\text{mH}}_{2} {\text{O}}$^14,15^ in which the ${\text{M}}^{2 + }$, ${\text{M}}^{3 + }$, and ${\text{A}}^{{{\text{n}} - }}$, are metals and charge balancing anion, respectively. These groups of inorganic layered nanomaterials have received scientists' attention because of their fantastic properties such as the ability to ion exchange and adsorption capacity. They can be easily designed to be used in the fields of catalysis^16^, environmental remediation^17,18^, sample preparation^19,20^, drug delivery^21^, and energy conversion and storage^22^. Agglomeration of nanosized materials such as LDHs is a well-known phenomenon as a result of their surface energy and characteristics. Their high surface area and polar or ionic surface make them thermodynamically unstable with respect to agglomerated species. These cause low efficiency for specific applications. To increase the performance and application field of LDHs, several modification strategies have been reported. Hybrid assembly, surface modification, intercalation, defect introduction, and layer composition tuning are some common functionalization strategies that can be found in detail in a review in 2020^23^.

Polymer matrix nanocomposites as a class of nanosized materials are becoming an inseparable part of various industries owing to their unique properties. Polymer matrix nanocomposites consist of a polymer or copolymer in which a nanosized filler is dispersed in the matrix of the polymer. The properties of a polymer matrix nanocomposite are related to the size scale of its components and the degree of mixing^24^. In this context, the use of nanosized filler in polymeric nanocomposites gathering the special advantages of nanosized filler including high surface area, and high thermal and mechanical stability, and the flexibility of organic polymers. Generally, the properties of nanocomposites are enhanced because of the ability of nanosized materials to affect the properties of the polymer matrix. The incorporation of layered nanosized fillers into the polymer matrices has attracted great attention because of their distinctive properties^25^. As a subclass of polymer/layered inorganic nanocomposites, LDHs-based polymer matrix nanocomposites have shown significant improvement in the composites’ overall physical and chemical properties. In the case of LDHs, various polymer matrix nanocomposites with organic polymers such as polyaniline, polypropylene, polyurethane, poly (vinylchloride), polyacrylonitrile, poly(methylmethacrylate), poly (ethylene terephthalate), etc. have been reported^26^. The use of natural polymers for the synthesis of polymer matrix nanocomposites has gained considerable interest to researchers in recent years. They are renewable, eco-friendly, biodegradable, and easily available. Among them, gellan gum polysaccharide produced by Sphingomonas elodea consists of glucose units, glucuronic acid, and rhamnose with a ratio of 2:1:1^27^.

In this work, a novel nanocomposite based on modified Cu-Ca-Al-LDH and gellan gum was synthesized for adsorption purposes. The LDH was synthesized with a simple and green route and then modified to improve compatibility with polymer matrix, surface area, adsorption properties, and other surface characteristics. The prepared material was then composited with Gellan gum as a natural polymer to obtain a novel polymer matrix nanocomposite-based adsorbent. To study the adsorption capability of the prepared polymer matrix nanocomposite, Congo red dye was chosen as a model analyte.

## Experimental section

### Materials and methods

In this study, copper(II) nitrate trihydrate (≥ 99.0%), calcium nitrate tetrahydrate (≥ 99.0%), aluminum nitrate nonahydrate (≥ 98.0%), sodium hydroxide (NaOH), sodium carbonate, ammonium persulfate, and itaconic acid were purchased from Sigma-Aldrich. Gellan gum, Congo red (CR), glacial acetic acid (99.5%), and phosphoric acid (85%) were purchased from Merck Co. A stock solution of Congo red was prepared in water at the concentration level of 2000 mg L^−1^.

### Apparatus and instruments

A Jasco-FT-IR-350 (Tokyo, Japan) instrument was applied (4400–400 cm^−1^, KBr pellets). Scanning and transmission electron microscopy (SEM, TEM) were done on a JSM-6510 (JEOL, Tokyo, Japan) and CM120 (Philips Electronics, Eindhoven, Netherlands) instruments, respectively. A Rigaku-DMax 2500 diffractometer (Japanese science and science Co., Tokyo, Japan) was used to record XRD patterns. N_2_ adsorption/desorption experiments were done on a Belsorp-mini II (BEL Japan Inc., Osaka, Japan) instrument. An STA 503 *(*Bahr GmbH*,* Hullhorst*,* Germany) instrument was used for thermogravimetric analysis (10 °C min^−1^, up to 800 °C). To determine dye concentration in sample solutions, a UV-1601 spectrophotometer (Shimadzu, Japan) was used (499 nm).

### Synthesis of the materials

This section aims to introduce a synthesis procedure to prepare itaconic acid-modified Cu–Ca–Al–LDH/gellan gum nanocomposites containing 1%, 3%, and 5% of filler. In a container, 8 mmol of Ca(NO_3_)_2_·4H_2_O (1908 mg), 8 mmol of Cu(NO_3_)_2_·3H_2_O (1952 mg), and 4 mmol Al(NO_3_)_3_·9H_2_O (1532 mg) were dissolved in 200 mL of pure water. After that, 200 mL of alkaline solution (50 mmol L^−1^ of Na_2_CO_3_ and 150 mmol L^−1^ of NaOH) was prepared in another container. The two solutions were mixed in an 800 mL glass flask (pH = 10–11 during the addition). After 60 min stirring at room temperature, the temperature of the mixture was increased to 60 °C and 100 mL of water with 5.0 g of itaconic acid was added to the mixture and maintained for 1 h. Then, 1 g of gellan gum to prepare nanocomposites containing 1%, 3%, and 5% of filler was dissolved in water (5.0 mL). This solution together with 1.0 mL of aqueous ammonium persulfate (0.065 mmol) was added to the LDH solution and stirred for 12 h. Then, the mixture was maintained for 20 h (without stirring). The obtained slurry was cooled, filtered, and washed with pure water and dried (85 °C).

### Batch adsorption experiments

For the experiments, 5.0 mL of an aqueous standard solution of CR was used. The adsorbent was added and the experiment was performed in a shaker (298 K, 220 rpm) for a period of time. To calculate the removal efficiency (RE, %), the following equation (Eq. 1) was used in which, *C*_*i*_ and *C*_*e*_ are the initial and equilibrium concentration of CR (mg L^−1^), respectively. To compute the adsorption capacity (q_e_, mg g^−1^), (Eq. 2) was applied in which, m is the amount of adsorbent (mg) and V is the volume of solution (mL).

1 $$ RE \left( \% \right) = \frac{{C_{i} - C_{e} }}{{C_{i} }} \times 100 $$

2 $$ q_{e} = \left( {\frac{{C_{i} - C_{e} }}{m}} \right) \times V $$

## Results and discussion

### Synthesis and characterization of the materials

**Figure 1:**
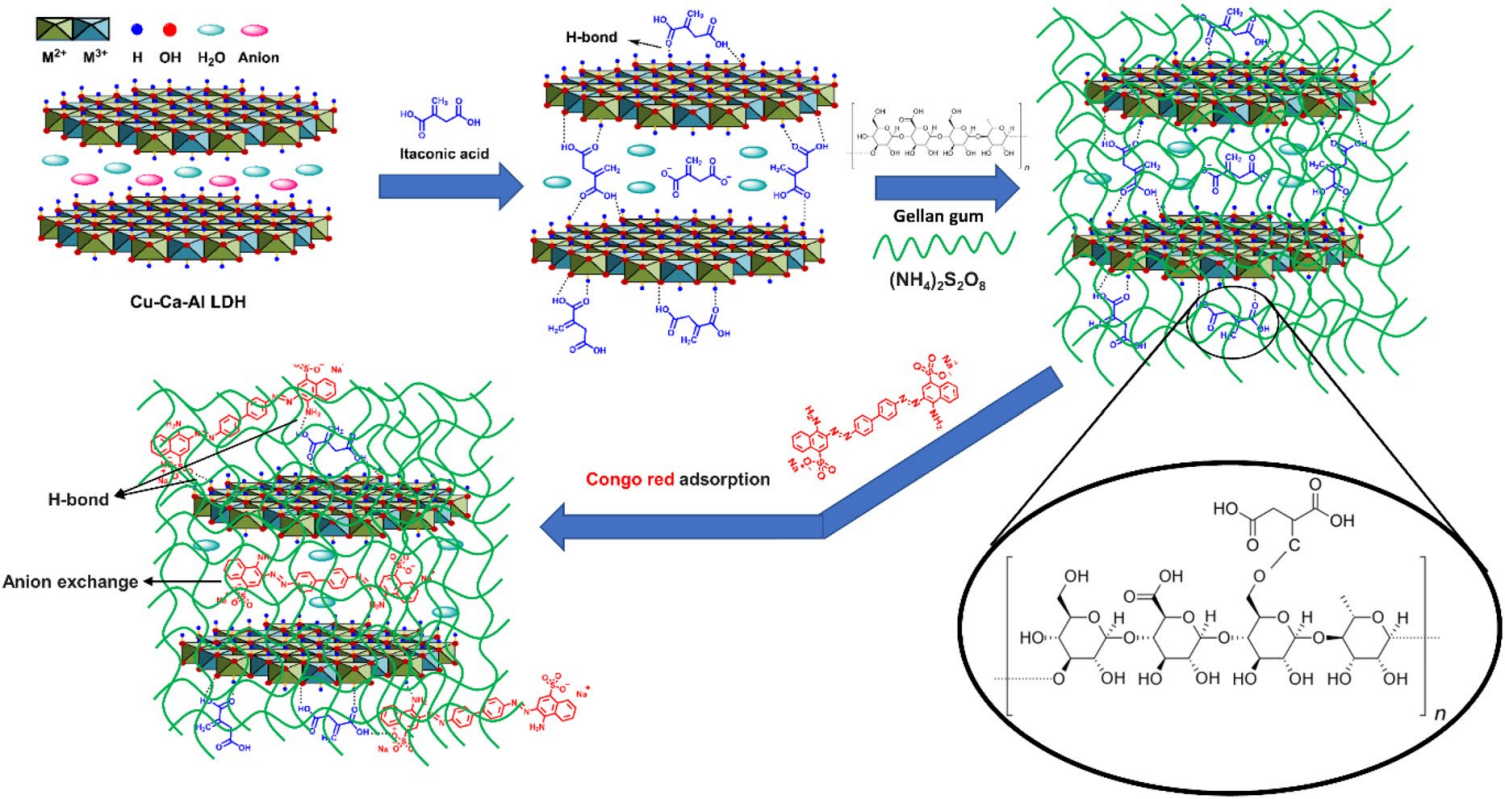
The schematic representation for the structure of the prepared adsorbents and their application for adsorption of CR from aqueous solutions.

In this work, a simple and green synthesis procedure was used for the preparation of novel modified Cu–Ca–Al–LDH/polymer matrix nanocomposites. At first, the LDH was simply prepared and then functionalized with itaconic acid. In this work, itaconic acid was applied as a surface modifier to decrease agglomeration and improve the surface area, compatibility with polymer matrix, and adsorption properties. The modified material was then incorporated into the natural polymer of gellan gum to produce polymer matrix nanocomposites with different filler contents. Water as a green and non-toxic solvent was used in the synthesis procedure (no use of hazardous solvents). The nanocomposites were used for the adsorption of Congo red from aqueous solutions due to their capability in hydrogen bond establishing between hydroxyl groups and functional groups of Congo red, as well as their ion-exchange ability. Figure 1 reveals the structure of the prepared materials and possible interactions during dye adsorption from an aqueous solution. As can be seen in Fig. 1, the itaconic acid as a modifier can establish a strong hydrogen bond with the surface of LDH. The hydrogen bond between hydroxyl groups and functional groups of Congo red and polymer matrix nanocomposite and also the anion-exchange mechanism are responsible for the adsorption process.

**Figure 2:**
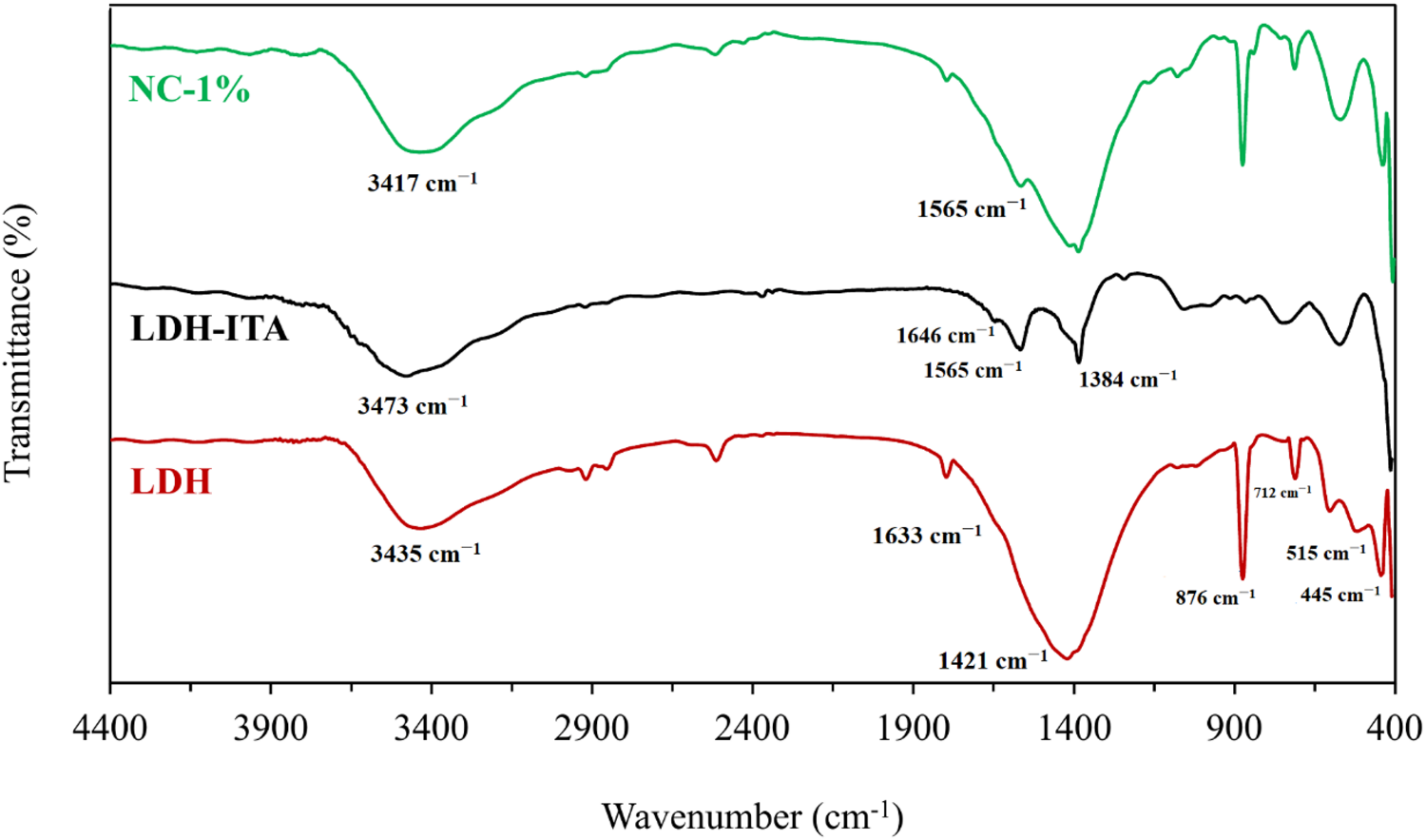
The FT-IR spectra of pristine LDH, LDH-ITA, and NC-1%.

The FT-IR spectra of Cu-Ca-Al-LDH, LDH-ITA, and nanocomposite containing 1% of filler (NC-1%) are shown in Fig. 2. The pristine Cu–Ca–Al–LDH provided broadband centered at 3435 cm^−1^ related to the O–H groups. On the other hand, the band at 1633 cm^−1^ is assigned to the water molecules bending vibration^28^. The band related to NO_3_^−^ anions of the interlayer can be seen at 1421 cm^−1^. Other bands including 876, 712, 604, 515, 445, etc., (vibrational modes of the LDH lattice) result from M–O–M, O–M–O, and M–O bonds (M = metal) ^29^. In the modified sample, the broadband centered at 3473 cm^−1^ is related to the hydroxyl groups. On the other hand, the bands at 1646 cm^−1^ and 1431 cm^−1^ are assigned to the carboxylic ions absorption bands^30,31^. The C=C stretching vibration can be observed at 1565 cm^−1^. The band located at 1421 cm^−1^ disappeared in LDH-ITA, indicating the replacement of NO_3_^−^ ions by itaconic acid anions. The band located at 1384 cm^−1^ is related to the carboxylate ions. In the case of NC-1%, in addition to the peaks related to the filler, the characteristic peaks of gellan gum also appear which is reported in previous work^27,32^. The broadband centered at 3417 cm^−1^ is related to the stretching vibrations of O–H (both gellan gum and filler). The C=C stretching vibration at 1565 cm^−1^ is weakened in NC-1% due to the reaction of radical of gellan gum with C=C bond of itaconic acid.

**Figure 3:**
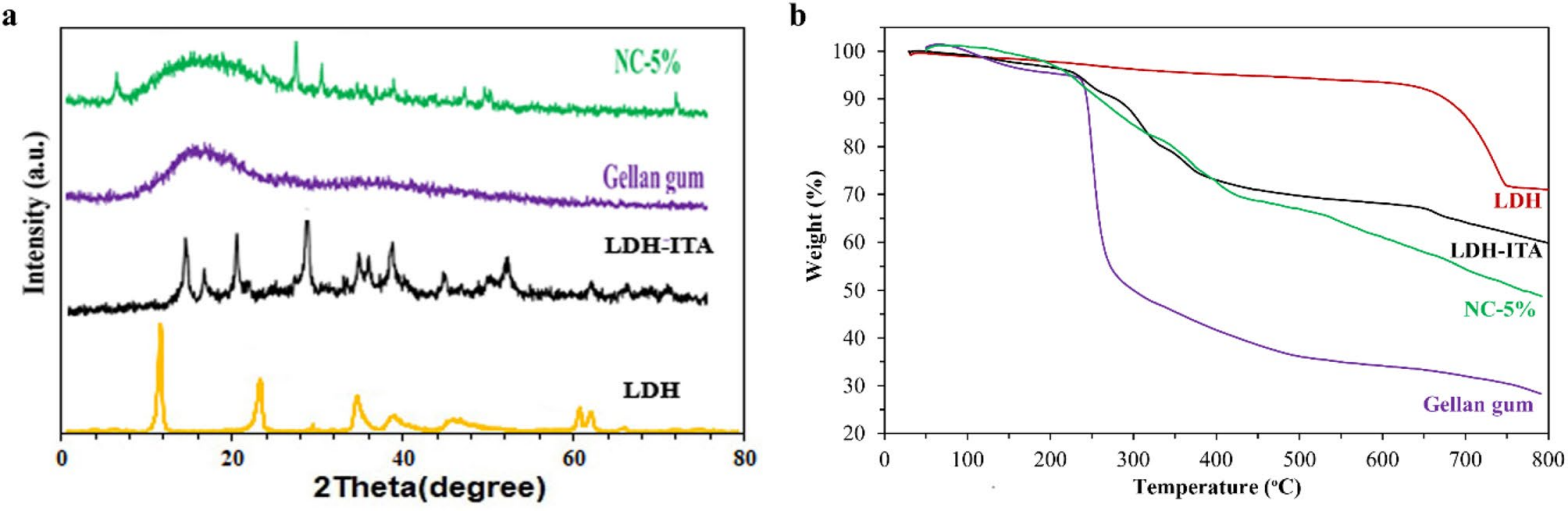
The XRD patterns (**a**) and the thermograms (**b**) of the prepared LDH, LDH-ITA, pure gellan gum, and NC-5%.

The XRD results of the materials are illustrated in Fig. 3a. The characteristic peaks of pristine Cu-Ca-Al-LDH are in accordance with previous reports^33^. For LDH-ITA, two peaks centered at 35.6° and 38.6° correspond to the CuO structure. The characteristic peaks of 23.4°, 29.5°, 36.9°, 39.5°, 43.1°, 47.5°, and 48.5° are related to CaCO_3_^33–35^. The disappearance of some diffraction peaks as well as appearing some new peaks in the LDH-ITA XRD pattern indicated incorporating the itaconic acid modifier into the LDH structure. In the case of gellan gum, broadband centered at 2θ values of 20 indicate its amorphous nature^27^. The NC-5% XRD pattern provided characteristic peaks related to LDH and amorphous gellan gum. The crystallinity and the amorphous characteristics of the prepared nanocomposites can be predicted from the sharp peaks and broad peak in the diffractogram.

The TGA curves of the prepared materials are illustrated in Fig. 3b. In the thermogram of the LDH, a mass loss (2.7%) was observed between 30 and 235 °C because of the removal of water^30^. Also, a mass loss of about 3.7% occurred between 235 and 580 °C due to decomposition of –OH groups and degradation of the layered structure. The final mass loss (580–800 °C, 22.6%) was occurred because of dehydroxylation of the LDH and the formation of metal oxides of calcium, copper, and aluminum. In the case of LDH-ITA, decomposition of itaconic acid began near 200 °C. The pure gellan gum showed degradation steps in its TGA curve. In the first step (below 150 °C), the moisture content of the sample is lost. In the second step (between 240 and 280 °C), a major weight loss of about 40% was occurred due to the breaking of the glycosidic linkage and decomposition of the polysaccharide backbone. Finally, decomposition of gellan gum occurred and only 28% of the sample remains as residual matter at 800 °C. The NC-5% showed several steps like LDH-ITA and provided better thermal stability in comparison to the pure polymer. More than 48% of the sample remains as residual matter at 800 °C.

**Figure 4:**
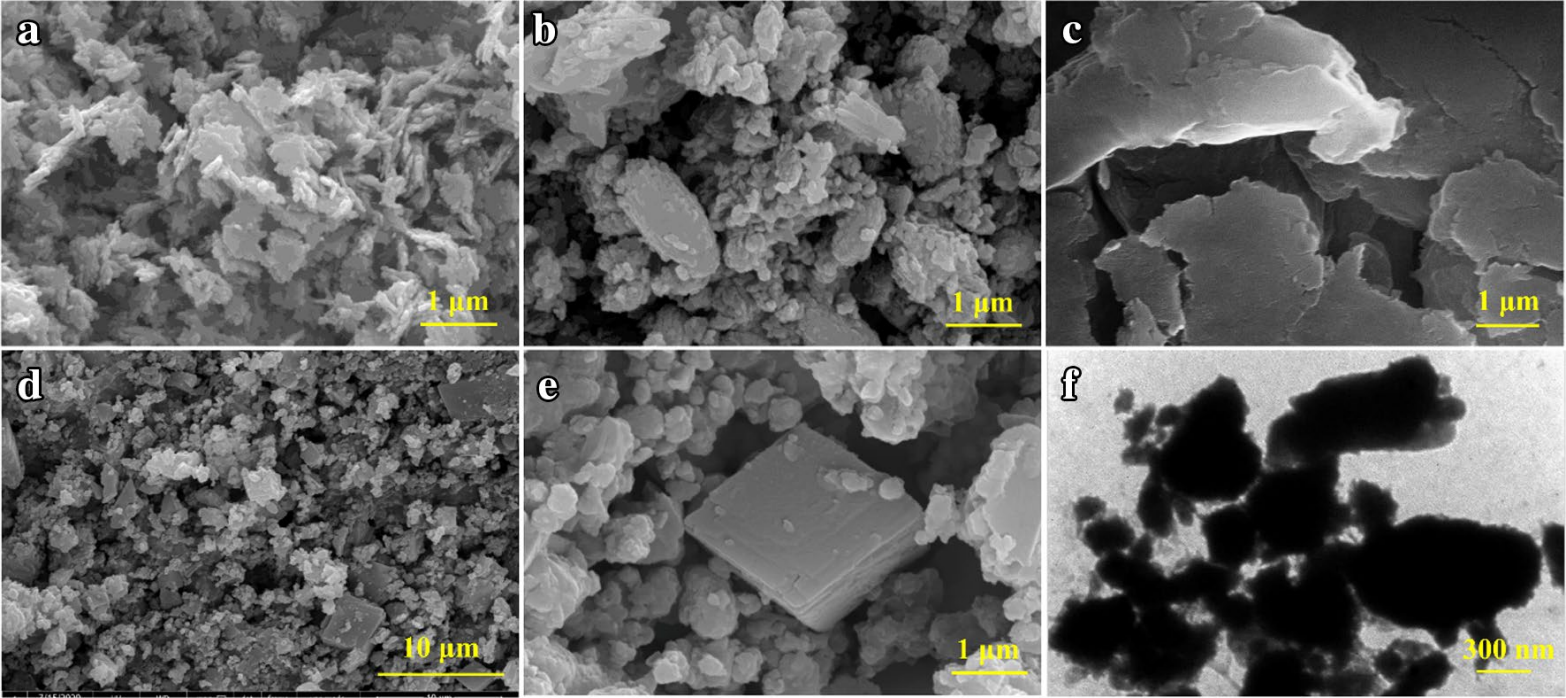
The SEM images of the prepared LDH (**a**), LDH-ITA (**b**), pure gellan gum (**c**), NC-1% (**d** and **e**), and the transmission electron micrograph of NC-1% (**f**).

The scanning and transmission electron microscopy images of the prepared materials are shown in Fig. 4a–f. The prepared LDH (Fig. 4a) and LDH-ITA (Fig. 4b) showed a plate-like morphology^33^. The pristine LDH represents a higher degree of aggregation in comparison with the modified LDH, because of hydrogen bond formation. In the case of pure gellan gum (Fig. 4c), a relatively homogeneous and smooth surface is observed^32^. As can be seen in Fig. 4d and e, the prepared NC-1% showed both homogeneous gellan gum particles and plate-like LDH-ITA filler. A relatively homogeneous dispersion of LDH-ITA in the polymer matrix appeared. The SEM and TEM images revealed a porous structure of the prepared materials to be used for adsorption purposes.

**Figure 5:**
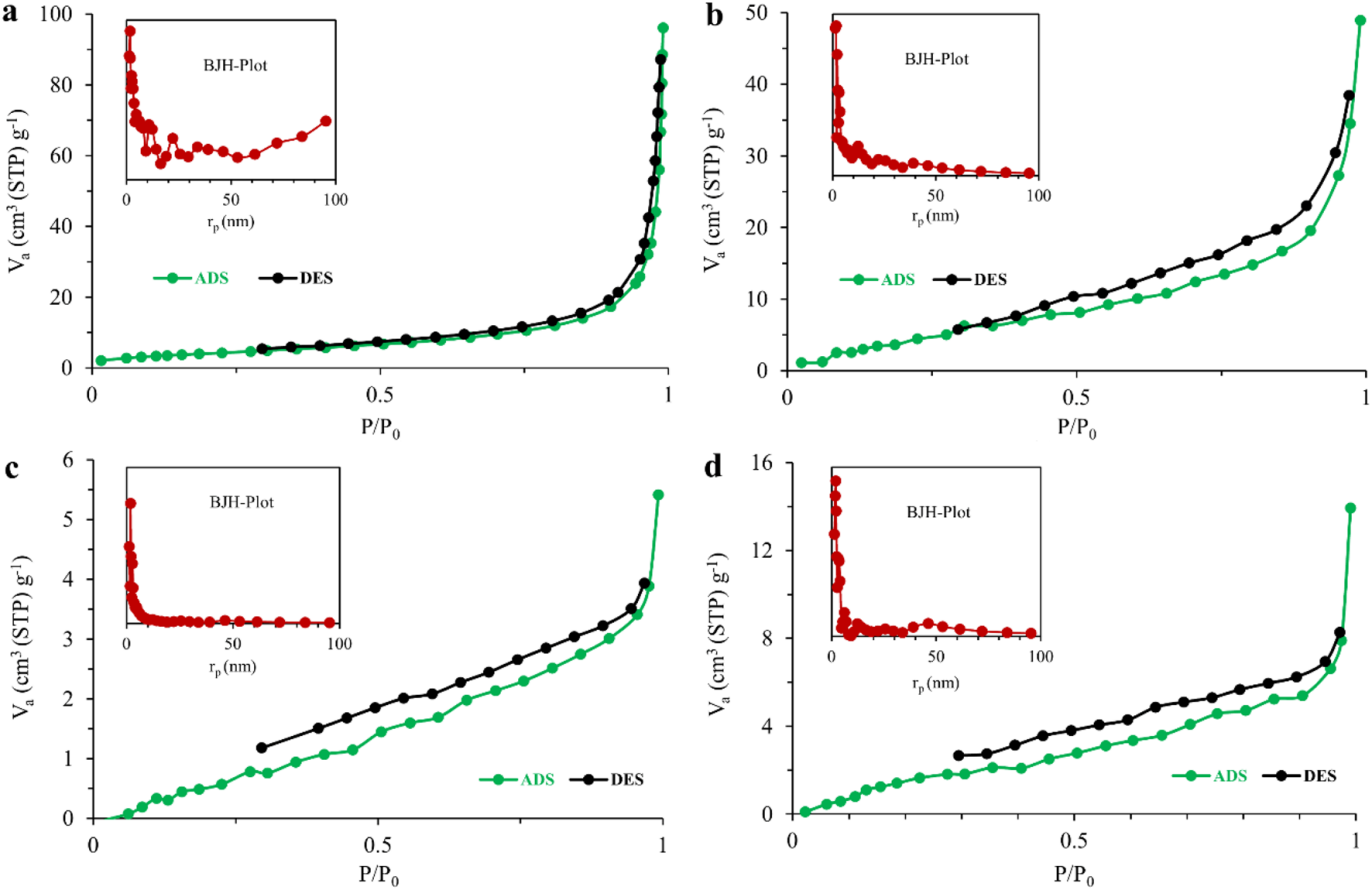
The N_2_ adsorption/desorption isotherms of LDH (**a**), LDH-ITA (**b**), pure gellan gum (**c**), and NC-1% (**d**).

The N_2_ adsorption/desorption isotherms of the materials are illustrated in Fig. 5. The pristine LDH provided a combination of isotherms of type III and IV (H3-type hysteresis loops). Also, the synthesized LDH-ITA exhibited IV-type isotherm (H3-type hysteresis loops). The prepared LDH and LDH-ITA showed BET surface areas of 15.6 and 24.8 m^2^ g^−1^ with pore volumes of 0.142 and 0.080 cm^3^ g^−1^, respectively (Table 1). Both pure gellan gum and NC-1% exhibited a combination of type III and IV isotherms and H2 and H3-type hysteresis loops. The flattened isotherms indicate less accessible pores in comparison with the prepared LDHs which are common in neat organic polymers. BET surface areas of 7.6 and 8.7 m^2^ g^−1^ with pore volumes of 0.0093 and 0.0223 cm^3^ g^−1^ were obtained for gellan gum and NC-1%, respectively (Table 1).

**Table 1:** Textural properties of the synthesized materials.

Sample	BET	BJH	
	Surface area (m^2^ g^−1^)	Vp (cm^3^ g^−1^)	rp (nm)
LDH	15.6	0.142	1.64
LDH-ITA	24.8	0.080	1.64
Gellan gum	7.6	0.0093	1.85
NC-1%	8.7	0.0223	1.85

### Adsorption studies

#### Sample solution pH

**Figure 6:**
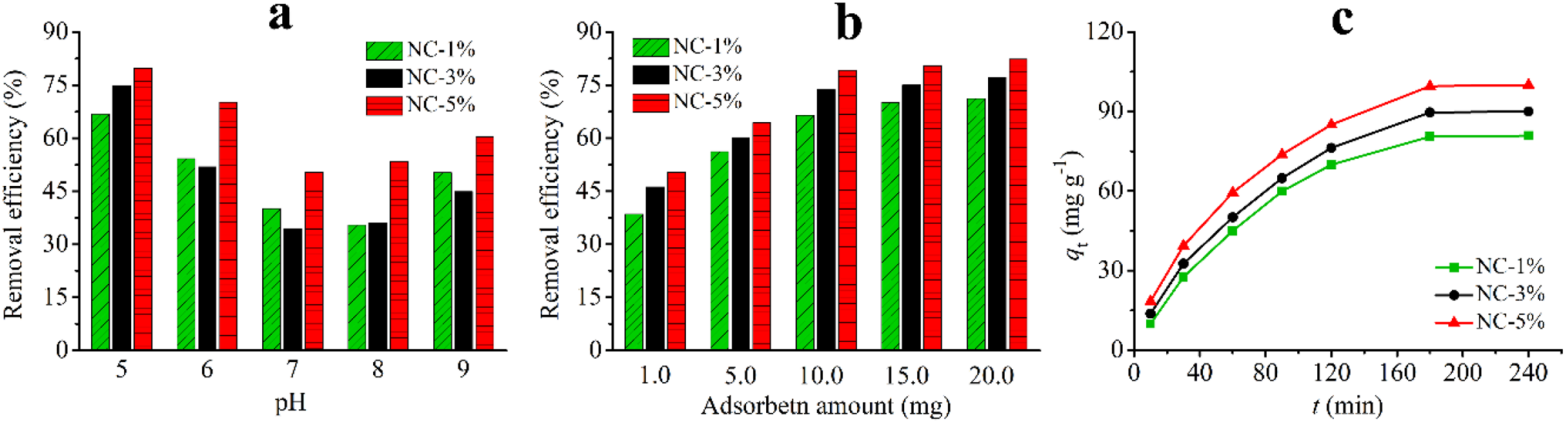
The effect of solution pH (**a**), adsorbent amount (**b**), and stirring time (**c**) on the adsorption of CR by the prepared nanocomposites.

The effect of sample solution pH was investigated between 5.0 and 9.0. Lower pHs were not studied because of the instability of the dye^36^. 5.0 mL of the standard solution of CR (50 mg L^−1^) and an adsorbent amount of 10.0 mg were used (298 K, 2 h, 1500 rpm). As Fig. 6a shows, the removal efficiency was firstly decreased with increasing the solution pH and then slightly increased. The decrease in the removal efficiency is may be related to this phenomenon that the surface of the material becomes less positive when pH enhanced from 5.0 to 7.0. Based on the results, pH = 5.0 was chosen.

#### Adsorbent amount

Various adsorbent amounts between 1.0 and 20.0 mg were tested. A dye concentration of 50 mg L^−1^ (pH = 5.0) was adopted (298 K, 2 h, 1500 rpm). The removal efficiency was first increased by enhancing the adsorbent dosage, and then no significant increase was observed in the case of all nanocomposites (Fig. 6b). Finally, 10.0 mg of each adsorbent was chosen.

#### Contact time

Contact times in the range of 10 and 240 min were tested to investigate the effect of time on the adsorption process. Standard solutions of CR (50 mg L^−1^, pH = 5.0) with an adsorbent amount of 10.0 mg were used (298 K). As Fig. 6c shows, an increase in removal efficiency occurred for all three nanocomposites when the stirring time was enhanced from 10 to 180 min. A time of 180 min was chosen for further experiments.

#### The kinetic studies

**Figure 7:**
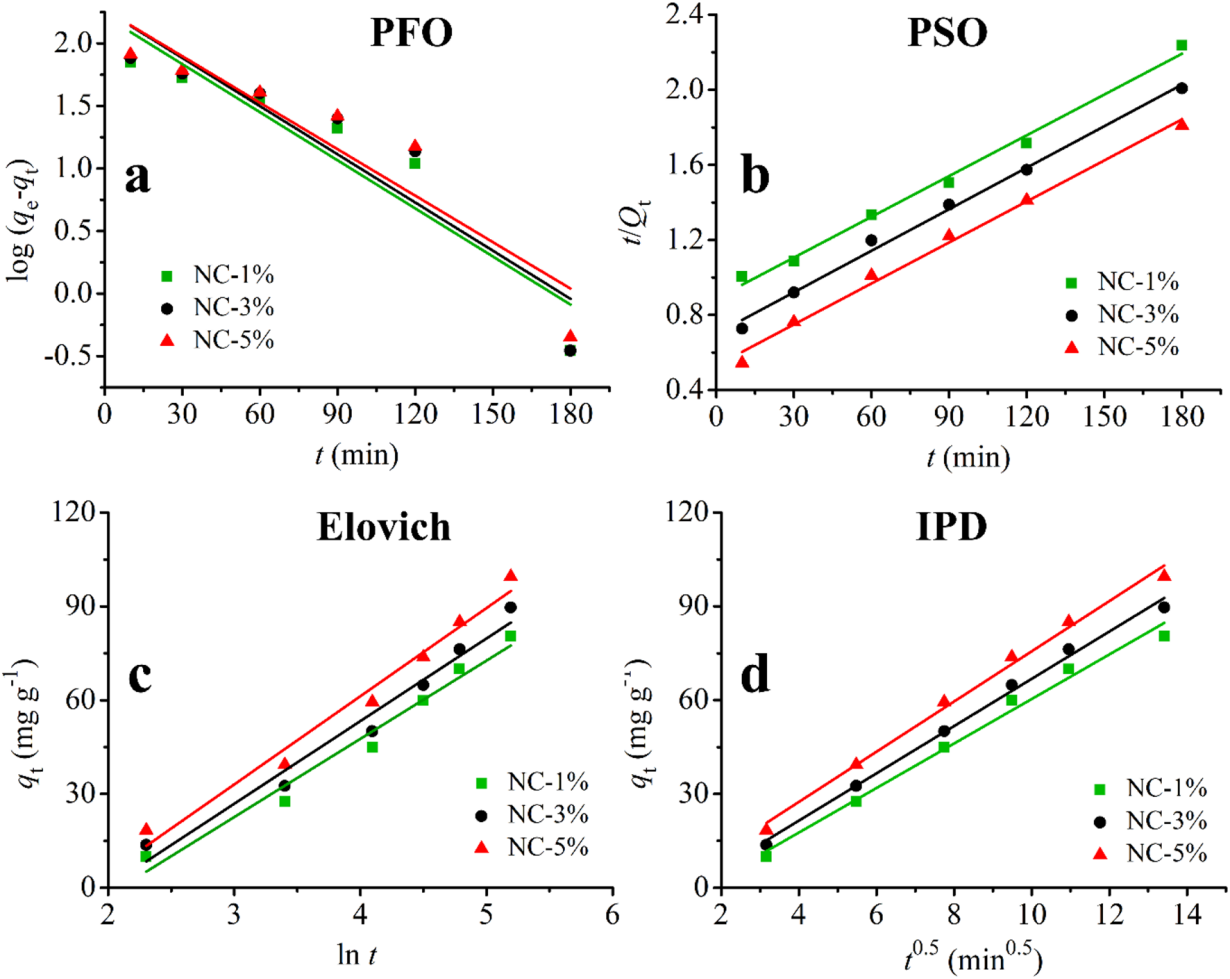
The pseudo-first-order (**a**), pseudo-second-order (**b**), Elovich (**c**), and intra-particle diffusion (**d**) adsorption models for the adsorption of CR.

In this step, four kinetic models including pseudo-first-order (PFO, Eq. 3), pseudo-second-order (PSO, Eqs. 4, 5), Elovich (Eq. 6), and intra-particle diffusion (IPD, Eq. 7) were used to study the mechanism of adsorption. In the following equations, *q*_*e*_ and *q*_*t*_ (mg g^−1^) are the capacity of adsorption at equilibrium condition and at time t, *k*_*1*_ (min^−1^) is PFO rate constant, *h* (mg g^−1^ min^−1^) is the initial sorption rate in PSO, *k*_*2*_ (g mg^-1^ min^−1^) is PSO rate constant, *α* (mg g^−1^ min^−1^) & *β* (g mg^−1^) are Elovich constants, *k*_*dif*_ (mg g^−1^ min^−0.5^) is the IPD rate constant. Also, *C* (mg g^−1^) is a constant. The obtained results are shown in Fig. 7 and Table 2. For all three nanocomposites, the PSO and IPD kinetic models provided the best results regarding the R^2^ value (a physiochemical process) with an intraparticle diffusion mechanism. The obtained R^2^ values for the PSO model were 0.9909, 0.9926, and 0.9906 for NC-1%, NC-3%, and NC-5%, respectively. Also, the obtained R^2^ values for IPD model were 0.9863, 0.9939, and 0.9918 for NC-1%, NC-3%, and NC-5%, respectively.

3 $$ \log \left( {q_{e} - q_{t} } \right) = \log q_{e} - \frac{{k_{1} }}{2.303}t $$

4 $$ \frac{t}{{q_{t} }} = \frac{1}{h} + \frac{1}{{q_{e} }}t $$

5 $$ h = k_{2} \times q_{e}^{2} $$

6 $$ q_{t} = \frac{{{\text{ln }}\left( {\alpha \beta } \right)}}{\beta } + \frac{\ln t}{\beta } $$

7 $$ q_{t} = k_{dif} (t)^{0.5} + C $$

**Table 2:** The kinetic parameters for the adsorption of Congo red onto the prepared nanocomposites.

Model	Adsorbent	*R*^2^	Parametersa		
PFO	NC-1%	0.8583	*k*_1_ = 2.95 × 10^–3^	*q*_*e*_ = 165.5	
	NC-3%	0.8272	*k*_1_ = 2.92 × 10^–3^	*q*_*e*_ = 185.6	
	NC-5%	0.8372	*k*_1_ = 2.85 × 10^–3^	*q*_*e*_ = 185.7	
PSO	NC-1%	0.9909	*k*_2_ = 5.83 × 10^–5^	*q*_*e*_ = 138.9	*h* = 1.1247
	NC-3%	0.9926	*k*_2_ = 7.83 × 10^–5^	*q*_*e*_ = 135.1	*h* = 1.4286
	NC-5%	0.9906	*k*_2_ = 1.01 × 10^–4^	*q*_*e*_ = 137.1	*h* = 1.8825
Elovich	NC-1%	0.9480	*α* = 3.098	*β* = 0.0399	
	NC-3%	0.9499	*α* = 3.652	*β* = 0.0378	
	NC-5%	0.9347	*α* = 4.559	*β* = 0.0354	
IPD	NC-1%	0.9863	*k*_*dif*_ = 7.1173	*C* = − 10.80	
	NC-3%	0.9939	*k*_*dif*_ = 7.5568	*C* = − 8.75	
	NC-5%	0.9918	*k*_*dif*_ = 8.0264	*C* = − 4.64	

^a^ The units are mentioned in “The kinetic studies”section.

#### Initial CR concentration and adsorption isotherm

**Figure 8:**
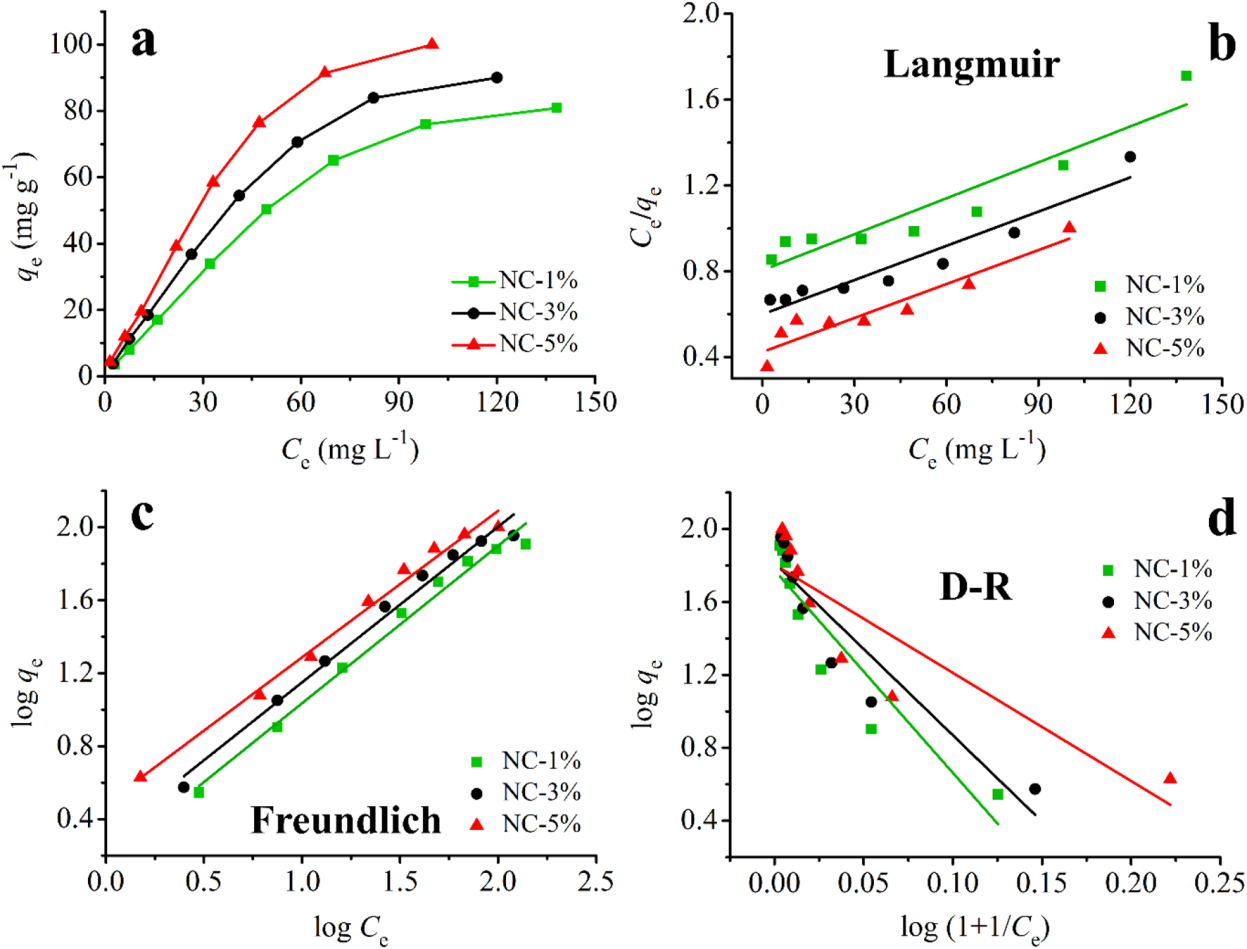
The equilibrium isotherm (**a**) and the isotherm models of Langmuir (**b**), Freundlich (**c**), and Dubinin–Radushkevich (**d**) for the adsorption of CR by the prepared nanocomposites.

In this step, various concentrations of CR (10–300 mg L^−1^, pH = 5.0) were tested (10 mg of adsorbent, 180 min contact time). Based on Fig. 8a, maximum adsorption capacities of 80.8, 90.1, and 99.9 mg g^−1^ were obtained for NC-1%, NC-3%, and NC-5%, respectively, for CR adsorption. Then, the Langmuir, Freundlich, and Dubinin–Radushkevich (D–R) isotherm models were studied. The used equations for Langmuir, Freundlich, and (D–R) isotherms are shown in Eqs. (8–10), respectively.

8 $$ \frac{{C_{e} }}{{q_{e} }} = \frac{1}{{q_{max} \times k_{L} }} + \frac{{C_{e} }}{{q_{max} }} $$

9 $$ \log q_{e} = \frac{1}{n}\log C_{e} + \log k_{F} $$

10 $$ \ln q_{e} = \ln q_{max} - B (RT\ln \left( {1 + \frac{1}{{C_{e} }}} \right))^{2} $$

In the above-mentioned equations, *C*_*e*_ (mg L^−1^) is the CR concentration at equilibrium, *q*_*e*_ (mg g^−1^) is the adsorption capacity at equilibrium, *q*_*max*_ (mg g^−1^) is the maximum adsorption capacity of the adsorbent, *k*_*L*_ (L mg^−1^) is the Langmuir model constant, *n* & *k*_*F*_ ((mg g^−1^) (L mg^−1^)^1/n^) are the Freundlich isotherm model constants, *B* (mol^2^ kJ^−2^) is the Dubinin–Radushkevich isotherm model constant, *R* (j mol^−1^ K^−1^) is the universal gas constant, and *T* (K) is temperature. The adsorption isotherms are illustrated in Fig. 8b–d. Also, the calculated parameters from the used isotherm models are illustrated in Table 3. Based on the obtained results and considering the R^2^ values, the Freundlich isotherm model showed the best fit for all three nanocomposites. The obtained R^2^ values were 0.9835, 0.9810, and 0.9868 for NC-1%, NC-3%, and NC-5%, respectively.

**Table 3:** The parameters of isotherm models for the adsorption of CR onto the prepared nanocomposites.

Model	Adsorbent	*R*^2^	Parameters^a^	
Langmuir	NC-1%	0.8780	*k*_*L*_ = 6.2 × 10^–3^	*q*_*max*_ = 200.0
	NC-3%	0.9090	*k*_*L*_ = 8.2 × 10^–3^	*q*_*max*_ = 204.1
	NC-5%	0.8811	*k*_*L*_ = 11.3 × 10^–3^	*q*_*max*_ = 208.3
Freundlich	NC-1%	0.9835	*k*_*F*_ = 1.479	*n* = 1.157
	NC-3%	0.9810	*k*_*F*_ = 1.972	*n* = 1.172
	NC-5%	0.9868	*k*_*F*_ = 3.041	*n* = 1.245
D–R	NC-1%	0.8579	*B* = 1.45 × 10^–7^	*q*_*ma*x_ = 59.4
	NC-3%	0.8388	*B* = 1.48 × 10^–5^	*q*_*max*_ = 65.3
	NC-5%	0.7464	*B* = 1.51 × 10^–5^	*q*_*max*_ = 68.3

^a^The units are mentioned in “Initial CR concentration and adsorption isotherm”section.

To study the reusability of the prepared material, the spent polymer matrix nanocomposites were washed with pure water while shaking for 5 min. After that, the adsorbent was separated from the solution by centrifugation and dried for further experiments. The regenerated adsorbent was subjected to repeated adsorption/desorption cycles. All the experiments were performed at optimum conditions. Data showed that the adsorbent can be reused 5 times with a 10% decrease in adsorption efficiency.

### Comparison with other studies

To compare the present method with other methods, contact time, pH, and adsorption capacity of the methods are provided in Table 4. The present method provided good adsorption capacity. Also, a shorter equilibrium time was obtained by the present method indicating the better performance of the prepared nanocomposites for CR removal due to the occurrence of adsorption through both anion exchange and surface adsorption by H-bonding mechanisms.

**Table 4:** Comparison of adsorption capacity, pH, and equilibrium time of the present method with other methods for removal of CR by reported adsorbents.

Adsorbentsa	qm (mg g^–1^)	pH	Equilibrium time (min)	Refernces
Cu-Ca-Al-LDH/gellan gum nanocomposite	74–100	5.0	60	This work
AuNPs-coated AC	71.05	6.5	270	^37^
AgNPs-coated AC	64.80	6.5	270	^37^
pTSA-Pani@GO-CNT	66.66	5.0	300	^38^
Neem leaf powder	41.24	6.7	300	^39^
Hollow ZnFe2O4 microspheres	16.58	6.0	120	^40^
Aspergillus niger biomass	14.16	6.0	1800	^41^
Modified Zeolite A	21.11	7.0	90	^42^
Montmorillonite	714.28	2.0	40	^43^
Kaolin	5.44	–	24 h	^44^

^*a*^NPs: nanoparticles; AC: activated carbon; *p*TSA: para toluene sulfonic acid; Pani: polyaniline; GO: graphene oxide; CNT: multiwalled carbon nanotube.

## Conclusions

In the present work, we synthesized novel modified Cu–Ca–Al–LDH/polymer matrix nanocomposites using a simple and environmentally friendly approach. The Cu–Ca–Al–LDH was synthesized at first and then functionalized with itaconic acid. The modified material was then incorporated into the natural polymer of gellan gum to produce polymer matrix nanocomposites with different filler contents. The FT-IR spectra, as well as TGA curves of the prepared materials, indicate the successful synthesis of the desired products. The XRD results showed characteristic peaks of the materials in accordance with previous reports. Also, the SEM and TEM images revealed the porous structure of the prepared materials. After the characterization of the synthesized nanocomposites, the prepared materials were used for the adsorption of CR from aqueous samples. The effect of pH, adsorbent dosage, stirring time, and dye concentration on adsorption was investigated. A sample pH = 5.0 with the adsorbent dosage of 10.0 mg and 180 min contact time were selected for isotherm and kinetic studies. The novel prepared materials provided notable adsorption performance toward CR. The PSO kinetic model obtained the best model for the adsorption of dye (R^2^ values of 0.9909, 0.9926, and 0.9906 for NC-1%, NC-3%, and NC-5%, respectively). In the case of isotherm models, the Freundlich model provided the best fit (R^2^ values of 0.9835, 0.9810, and 0.9868 for NC-1%, NC-3%, and NC-5%, respectively). Maximum adsorption capacities of 74, 80, and 100 mg g^-1^ were obtained for NC-1%, NC-3%, and NC-5%, respectively, for CR adsorption. Prospects related to this class of adsorbent include the synthesis of polymer matrix nanocomposites utilizing other high-performance nanosized materials to improve adsorption capability and also tuning the sorbent structure for selective adsorption.
